# The Liver Maximum Capacity Test (LiMAx) Is Associated with Short-Term Survival in Patients with Early Stage HCC Undergoing Transarterial Treatment

**DOI:** 10.3390/cancers14215323

**Published:** 2022-10-28

**Authors:** Janett Fischer, Stella Wellhöner, Sebastian Ebel, Thomas Lincke, Albrecht Böhlig, Florian Gerhardt, Rhea Veelken, Holger Goessmann, Karen Geva Steinhoff, Timm Denecke, Osama Sabri, Thomas Berg, Florian van Bömmel

**Affiliations:** 1 Division of Hepatology, Department of Medicine II, Leipzig University Medical Center, 04103 Leipzig, Germany; 2 Department of Diagnostic and Interventional Radiology, Leipzig University Medical Center, 04103 Leipzig, Germany; 3 Department of Nuclear Medicine, Leipzig University Medical Center, 04103 Leipzig, Germany

**Keywords:** TACE, TARE, liver function, adverse events, survival

## Abstract

**Simple Summary:**

The liver maximum capacity test (LiMAx) represents a useful tool to estimate liver function in patients with chronic liver disease. LiMAx results correlate with short-term survival in patients with early stage HCC after transarterial chemo- or radioembolization. Low LiMAx levels might enable the identification of patients with poor hepatic function and decreased short-term survival after treatment.

**Abstract:**

Transarterial chemoembolization (TACE) and transarterial radioembolization (TARE) are recommended to treat patients with early or intermediate hepatocellular carcinoma (HCC). The liver maximum capacity test (LiMAx) has been supposed to predict the risk of post-interventional liver failure. We investigated the correlation of LiMAx with short-term survival as primary endpoint and the occurrence of adverse events after therapy as secondary endpoint. Our study cohort prospectively included 69 patients receiving TACE (n = 57) or TARE (n = 12). LiMAx test and serological analyses were performed on the day before and 4 weeks after treatment. Hepatic and extrahepatic complications were monitored for 4 weeks. The LiMAx results were not associated with altered liver function and the occurrence of adverse events. The survival rates of patients with BCLC A with LiMAx ≤ 150 μg/kg/h were lower after 30 days (75.0 ± 15.3% vs. 100%, *p* = 0.011), 90 days (62.5 ± 17.7% vs. 95.8 ± 4.1%, *p* = 0.011) and 180 days (50.0 ± 17.7% vs. 95.8 ± 4.1%, *p* = 0.001) compared to those with higher LiMAx levels. The LiMAx test is not suitable to predict liver function abnormalities or the occurrence of complications 4 weeks after therapy but enables the identification of patients with early stage HCC and reduced short-term survival after treatment.

## 1. Introduction

Hepatocellular carcinoma (HCC) is the sixth most common cancer and the third leading cause of cancer-related death worldwide [1]. Transarterial chemoembolization (TACE) is a widely used first-line therapy for treatment of unresectable HCC in patients with early or intermediate-stage disease according to the Barcelona Clinic Liver Cancer Classification (BCLC) [2,3]. Transarterial radioembolization (TARE) has been proposed as an effective alternative to TACE, and it is the most common treatment option for patients with locally advanced HCC [2,3,4,5,6]. Both transarterial treatment strategies can help control local tumor growth, reduce palliate symptoms, prolong survival, or bridge the time to liver transplantation [7,8].

However, in HCC patients the individual prognosis largely depends on liver function. This is of special interest as the majority of HCC patients have underlying liver cirrhosis, and thus inadequate hepatic function increases the risk of severe complications and hepatic decompensation after TACE or TARE [8,9]. Accordingly, acute hepatic failure, which can occur in 3–5% of patients, is one of the most serious complications after TACE [10,11,12,13].

Predicting the outcome of a transarterial treatment is a high medical need, as systemic treatments are increasingly becoming available, which could potentially have a greater benefit for the patient in the context of a personalized treatment concept. To this end, algorithms have been developed that assess the risks inherent in transarterial treatments [2,14,15,16,17]. Thus, increased serum bilirubin levels and severe Child–Turcotte–Pugh (CTP) stages are considered risk factors for liver failure after TACE [18]. The Cancer of the Italian Liver Program (CLIP) Score was established, which included the CTP classification system and several aspects of tumor propagation [19]. Furthermore, the model for end-stage liver disease (MELD) and albumin–bilirubin (ALBI) scores have been shown to be potent predictors of post-therapeutic outcome and overall survival [14,20,21,22,23]. However, such scores depend on laboratory parameters and the subjective estimation of clinical symptoms. Therefore, direct measurement of liver function might be a superior method to predict tolerability of transarterial treatment approaches.

The liver maximum capacity test (LiMAx, Humedics, Berlin, Germany) is a dynamic liver function bedside test, which provides a comparable and quantitative value of enzymatic liver function capacity. The test assesses metabolism of intravenously injected 13C-methacetin by a liver-specific cytochrome P450 1A2-system. In several previous studies, LiMAx was successfully evaluated in patients with different stages of liver fibrosis [24,25], with acute liver failure [26] and with bacterial sepsis [27,28]. This tool is potentially suitable to select candidates for liver surgery or liver transplantation and to predict the post-operative outcome [29,30,31]. Recent studies in small patient populations also showed that LiMAx might be an appropriate monitoring tool to predict the risk of liver failure after TACE [32,33,34].

The primary aim of our study was to investigate the correlation of LiMAx results with short-term survival in patients with early and intermediate stage HCC after TACE or TARE. The secondary aim was to assess the potential to predict the occurrence of adverse effects and liver deterioration four weeks after transarterial treatment.

## 2. Patients and Methods

### 2.1. Patients and Study Design

Our study was conducted on patients who underwent TACE or TARE between November 2017 and April 2020 the University Medical Center. Patients were successively included into the study by availability. A multidisciplinary HCC tumor board made the decision to perform TACE or TARE in the enrolled patients. Liver function was assessed on the day before as well as at 4 weeks after TACE or TARE procedures using the LiMAx test and the well-established serological analyses. Our study was approved by the Ethics Committees of Medical Research of the University of Leipzig (vote no. 213/17-ek) in accordance with the Declaration of Helsinki from 1975 (revision 2013) and the International Conference on Harmonization/Committee for Proprietary Medicinal Products’ “Good Clinical Practice” guidelines. All patients provided written informed consent. The following data were prospectively collected: patient demographic and laboratory data, cancer characteristics, CTP score, MELD score, ALBI score, and LiMAx results, as well as the occurrence of adverse effects of treatment at 4 weeks after TACE/TARE, and survival until 34 month after treatment. Adverse events of treatment were categorized according to the Society of Interventional Radiology (SIR) Adverse Event Classification in (a) mild: no or nominal therapy; (b) moderate: modest escalation of care, requiring intervention, extremely prolonged outpatient observation or overnight admission after outpatient procedure; (c) severe: marked escalation of care or complex intervention; (d) life-threatening or disabling event, e.g., cardiopulmonary arrest, shock, organ failure, unanticipated dialysis, paralysis, loss of limb or organ; and (e) patients death [13]. Furthermore, the occurrence of REILD was assessed and considered as classic with symptoms of fatigue, abdominal pain, increased abdominal girth, hepatomegaly, anicteric ascites 1–3 months after TARE, and a twofold increase of the alkaline phosphatase; or non-classic with dysregulated hepatic functions with jaundice and/or remarkably elevated serum transaminases [35].

### 2.2. Transarterial Chemoembolization (TACE)

All TACE procedures were routinely performed in the clinic using a standard protocol consisting of doxorubicin, mitomycin C, and lipiodol. According to the guideline, a coaxial 2.7 French microcatheter was placed into the hepatic artery to selectively visualize the tumor vessels. Then, doxorubicin, mitomycin C and lipiodol were selectively applied to the tumor vessels. Several weeks after TACE, all patients received a local computer tomography scan to evaluate the embolized liver volume. TACE was performed based on interdisciplinary tumor board decision as palliative treatment or as bridging treatment before liver transplantation.

### 2.3. Transarterial Radioembolization (TARE)

After diagnostic angiography and Tc-99m-MAA scintigraphy for treatment planning, a mean dose of 2.28 ± 1.20 [median 1.96 (range 0.7–4.3)] Giga-Becquerel (GBq) yttrium-90 glass microspheres (TheraSphere, Boston Scientific, Marlborough, MA, USA) was manually injected through a microcatheter. The distribution of the microspheres in the tumor was recorded 24 h after treatment via single-photon emission computed tomography.

### 2.4. LiMAx

The LiMAx test (Humedics GmbH, Berlin, Germany) was performed after a minimum of 3 h fasting. As previously described, the test procedure is based on intravenous administration of 2 mg/kg body weight ^13^C-methacetin, which is selective substrate of the hepatic cytochrome P450 1A2 enzyme [30]. The liver specific enzyme demethylates ^13^C-methacetin into acetaminophen and ^13^CO_2_, which is subsequently exhaled. The ratio of ^13^CO_2_/^12^CO_2_ concentration was constantly monitored online in the exhaled breath over a period of 60 min maximum using an infrared absorption spectroscopy method. The baseline ratio of ^13^CO_2_/^12^CO_2_ concentration was recorded in the native exhaled air before substrate injection. LiMAx value was calculated according to the previously described formula [30]. Results are given in μg/kg/h and available directly after test termination. The LiMAx values > 315 μg/kg/h were considered normal [36].

### 2.5. Statistical Analysis

Statistical analyses of epidemiological associations were performed using SPSS software (SPSS Inc., version 25.0, Chicago, IL, USA). Values are presented in median and interquartile range if not otherwise specified. Categorical variables are shown as frequencies and percentage. The Chi-squared test was applied for categorical variables and the Mann–Whitney U-test and Wilcoxon signed-rank test to compare quantitative variables. Correlations were calculated using the Spearman correlation coefficient. Univariate and multivariate logistic regression analyses (inclusion model) were used to determine the association between different parameters. The regression coefficient (RC), standard error (SE), odds ratio (OR) and the 95% confidence interval (CI) were calculated. Survival analyses were performed with Kaplan–Meier estimator and Cox regression analysis for 30-, 60-, 90-, and 180-day survival as well as for overall survival. Multivariate regression analysis was performed by using *p* < 0.05 for inclusion and *p* > 0.1 for exclusion of parameters in the final model. All tests were two-sided and *p* values of <0.05 were considered significant.

The CTP score is based on total serum bilirubin and albumin and the international normalized ratio for prothrombin time (INR) as wells on the quantification of the severity of ascites and hepatic encephalopathy from none to mild to severe [37,38]. Patients were classified in Child A with CTP points 5–6 and in Child B with CTP points 7–9. The MELD sore included the serum levels of bilirubin and creatinine and INR, and is calculated according to the formula: MELD = 3.78 × ln (serum bilirubin [md/dL]) + 11.2 × ln (INR) + 9.57 × ln (serum creatinine [mg/dL]) + 6.43 [39].

The ALBI score was calculated as previously described [23]: (log_10_ bilirubin [mmol/L] × 0.66) + (albumin [g/L] × (−0.0852)). ALBI classes were determined as follows: ALBI score ≤ −2.60 (ALBI grade 1), −2.60 to ≤−1.39 (ALBI grade 2), and ≥−1.39 (ALBI grade 3) [40]. Patients were divided into two groups by a LiMAx cut-off of 150 μg/kg/h before transarterial treatment that was previously identified to be associated with worse outcome [29].

## 3. Results

### 3.1. Study Population

**Figure 1:**
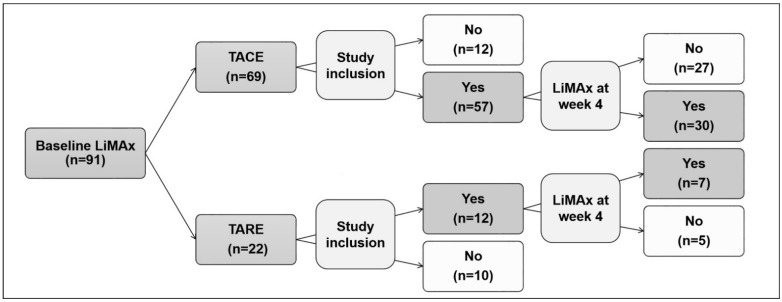
Patients included in the present study. A total of 12 patients with TACE and 10 patients with TARE were excluded because of concomitant cancer diseases such as cholangiocarcinoma, colon carcinoma, and bile duct carcinoma or other contraindications as well as missing data sets. LiMAx: liver maximum capacity test, TACE: transarterial chemoembolization TARE: transarterial radioembolization.

LiMAx was performed in 91 patients between November 2017 and April 2020. In total, 22 patients were excluded because of concomitant cancer diseases such as cholangiocarcinoma (n = 3), colon carcinoma (n = 2), and bile duct carcinoma (n = 2), other carcinoma (n = 1), as well as missing data sets (n = 4). TACE or TACE was cancelled in 10 patients after evaluation because of contraindications such as arteriovenous shunts, metastases, coronary diseases, or consent withdrawal (Figure 1).

Our study cohort included consecutive 69 patients of whom 57 (82.6%) patients were treated with TACE and 12 (17.4%) with TARE. Patients’ characteristics are summarized in Table 1, which also demonstrates the similar characteristics of both groups. Alcoholic liver disease was the main cause of liver cirrhosis (60.3%), and the majority of patients presented a liver cirrhosis with CTP Child A (68.1%). In the cohort, 48.5% of patients had ALBI grade 1 and 48.5% had ALBI grade 2. Two patients had ALBI grade 3. The median MELD score was 8 (6–20) points. BCLC stages A, B and C were present in 32 (46.4%), 29 (42.0%), and 8 (11.6%) patients. There were no significant differences between the two treatment groups. Two (2.9%) patients died within 4 weeks after TACE and one patient was lost to follow up.

### 3.2. LiMAx Results and Other Parameters of Liver Function before and after Transarterial Treatment

**Figure 2:**
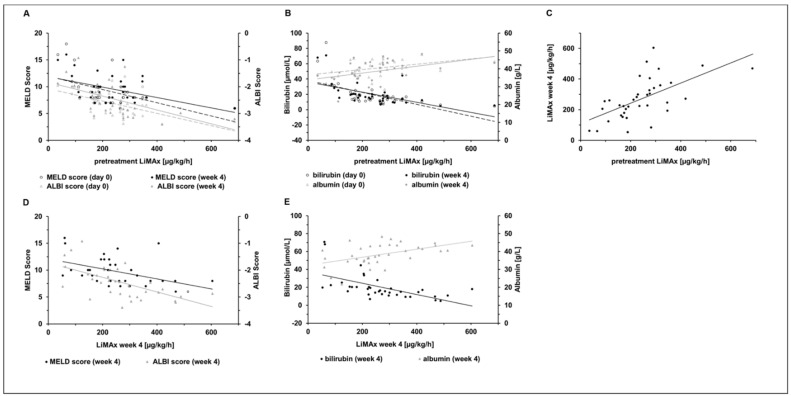
Correlation of (**A**) baseline LiMAx levels with MELD and ALBI scores and (**B**) baseline LiMAx levels with serum bilirubin and albumin before treatment (day 0) and after treatment (week 4), of (**C**) baseline LiMAx with LiMAx levels at week 4 and of LiMAx levels with (**D**) MELD and ALBI score and (**E**) bilirubin and albumin at week 4. ALBI: albumin–bilirubin, LiMAx: liver maximum function test MELD: model for end-stage liver disease.

LiMAx was assessed in 69 patients before and in 37 (53.6%) patients at week 4 after transarterial treatment. Before transarterial treatment, LiMAx results showed intermediate correlation with bilirubin (r = −0.569, *p* = 0.0004) albumin (r = 0.399, *p* = 0.016), with AST (r = −0.490, *p* = 0.002) and with INR (r = −0.365, *p* = 0.026). LiMAx results showed intermediate correlation with ALBI score (r = −0.569, *p* = 0.0003 and MELD score (r = −0.504, *p* = 0.002) (Figure 2).

There were no significant changes in LiMAx levels and blood parameters between the day before and 4 weeks after transarterial treatment (Table 2). The MELD score, ALBI score and CTP score also did not significantly differ between the two time points. Interestingly, the LiMAx results before treatment correlated with the LiMAx results (r = 0.609, *p* = 6.23 × 10^−5^), ALBI score (r = −0.421, p = 0.0004) and MELD score (r = 0.421, *p* = 0.013), bilirubin (r = −0.562, *p* = 0.0003) and albumin (r = 0.393, *p* = 0.016) at week 4 after transarterial treatment.

After transarterial treatment, LiMAx results correlated with the liver function scores CPT (r = −0.362, *p* = 0.036), MELD (r = −0.462, *p* = 0.006) and ALBI (r = −0.618, *p* = −0.618, *p* = 4.65 × 10^−5^) and with bilirubin (r = −0.576, *p* = 0.0002) and albumin (r = 0.493, *p* = 0.002) (Figure 2).

When the study cohort is stratified according to CTP and ABLI grade groups, significant differences in LiMAx were observed in the subgroups. The ALBI grade 1 group showed higher LiMAx levels than ALBI grade 2/3 before (median 276 (range 156–686) µg/kg/h vs. median 173 (range 35–282) µg/kg/h, *p* = 0.0001) and after TACE/TARE (median 316 (range 53–604) µg/kg/h vs. median 192 (range 60–283) µg/kg/h, *p* = 0.0002). Furthermore, a significant increase in LiMAx levels after treatment was detected in the Child A group (median 229 (range 87–686) µg/kg/h vs. median 261 (range 63–604) µg/kg/h, *p* = 0.018) but not in Child B nor in the ALBI grade subgroups (Supplementary Tables S1 and S2).

When we compared patients with lower LiMAx levels (LiMAx ≤ 150 μg/kg/h) versus those with higher LiMAx levels (LiMAx > 150 μg/kg/h), significant differences in blood parameters and liver function scores were assessed. Patients with LiMAx ≤ 150 μg/kg/h showed significantly increased levels of liver enzymes and increased ALBI, MELD, and CTP scores, as well as decreased levels of albumin and platelets counts compared to patients with higher LiMAx levels before and after treatment. However, within both LiMAx groups no significant differences were observed in almost parameters but ALBI score (median −2.83 (range −3.40–−1.61)) vs. median −2.71 (range −3.3–−1.25) *p* = 0.003) and albumin levels (median 41.0 (range 28.5–48.0) g/L vs. median 40.6 (range −25.2–48.3) g/L *p* = 0.007) in the LiMAx > 150 µg/kg/h group before and 4 weeks after TACE/TARE (Supplementary Table S3).

### 3.3. LiMAx Results and Adverse Events of Transarterial Treatment

Common adverse events were recorded for 4 weeks after treatment, which are summarized in Supplementary Table S4. Only 27 patients (39.1%) showed mild extrahepatic adverse events such as fatigue (11.6%) and epigastric pressure (7.2%) in both treatment groups. In the TACE group, 15.8% of patients suffered from severe complications related to liver dysfunction such as ascites and hepatic encephalopathy requiring intervention. Two patients developed a kidney failure after treatment, and one patient showed a myocardial infarct. This patient had CTP Child A, MELD 17, ALBI grade 3 and BCLC A stage and a LiMAx level of 129 μg/kg/h before TACE and died 3 days after treatment because of acute-on-chronic liver failure (ACLF). Another patient also died due to ACLF within 4 weeks after treatment. In the TARE group, one patient with BCLC C stage developed symptoms of a non-classical REILD with jaundice and mild ascites as well as an increase in CPT score (from 5 to 9), MELD score (from 12 to 17) and in ALBI grade (from 1 to 3) 4 weeks after treatment, and died 71 days after therapy.

Overall, there was no significant association between the occurrence of common adverse events and LiMAx levels before TACE/TARE (*p* = 0.155). In contrast, the incidence of complications was associated with the liver function scores before treatment: CTP score (OR = 2.15 [95% CI: 1.07–4.33] *p* = 0.032) and ALBI score (OR = 2.73 [95% CI: 1.01–7.40] *p* = 0.048) and AST levels (OR = 5.83 [95% CI: 1.08–31.98] *p* = 0.040) in univariate logistic regression analysis. However, in multivariate regression analysis no independent factor was identified (Supplementary Table S5).

### 3.4. Association of LiMAx Results with Survival

The median survival time was 16 (range 0–34) month. Survival analyses of both treatment arms and LiMAx groups (≤150 μg/kg/h and >150 μg/kg/h) were performed after 30, 60, 90, and 180 days as well as after 34 month for overall survival.

The median survival in the TARE group was 12 (range 2–19) months, and in the TACE group, it was 18 (range 0–34) months (*p* = 0.033). However, there were no significant differences in survival rates between TARE and TACE treatment in the 30-day (100% vs. 96.5 ± 2.4%, *p* = 0.515), 60-day (100% vs. 94.7 ± 3.0%, *p* = 0.423), 90-day (91.7 ± 8.0% vs. 91.2 ± 3.7%, *p* = 0.942), and 180-day (83.3 ± 10.8% vs. 80.7 ± 5.2%, *p* = 0.845) and overall (46.9 ± 17.6% vs. 59.6 ± 7.3%, *p* = 0.279) survival.

**Figure 3:**
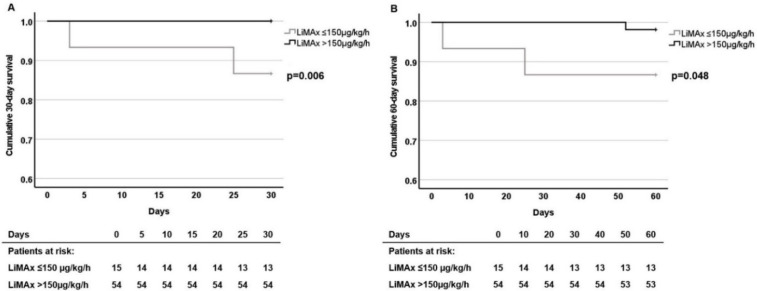
Survival rates in the total cohort according to the LiMAx ≤ 150 μg/kg/h and LiMAx > 150 μg/kg/h groups: (**A**) 30 days and (**B**) 60 days after transarterial treatment. Survival analyses were performed with Kaplan-Meier estimator.

Patients with LiMAx results ≤ 150 μg/kg/h revealed significant lower 30-day and 60-day survival rates (SR) compared to patients with LiMAx > 150 μg/kg/h levels (30-day SR: 86.7 ± 8.8% vs. 100%, *p* = 0.006; 60-days SR: 86.7 ± 8.8% vs. 98.1 ± 1.8%, *p* = 0.048, Figure 3). After 90 and 180 days, the differences in survival rates between the two patient groups were indistinct (90 days: 80.0 ± 10.0% vs. 94.4 ± 3.1%, *p* = 0.070; 180 days: 73.3 ± 11.4% vs. 83.3 ± 5.1%, *p* = 0.318). The overall survival rates were not different between the two groups (*p* = 0.239). The median (range) survival of patients with LiMAx results ≤ 150 μg/kg/h was 18 (0–33) month and of patients with LiMAx results > 150 μg/kg/h was 16 (2–34) month (*p* = 0.844).

In univariate Cox regression analysis, a reduced overall survival was associated with increased levels of bilirubin (Hazard ratio (HR) = 1.03 [95% CI: 1.01–1.05] *p* = 0.002), decreased albumin levels (HR = 0.89 [95% CI: 0.82–0.97] *p* = 0.005), as wells as increased MELD (HR = 1.17 [95% CI: 1.05–1.32] *p* = 0.005), ALBI score grade 2 (HR = 2.58 [95% CI: 1.05–6.34) *p* = 0.038) and grade 3 (HR = 50.43 [95% CI: 8.12–313.06] *p* = 2.57 × 10^−5^), and CTP Child B (HR = 2.93 [95% CI: 1.27–6.76] *p* = 0.012) before treatment. In multivariate Cox regression analysis, MELD and ALBI grade 3 remained predictors for decreased overall survival with a HR of 1.15 (95% CI: 1.00–1.33, *p* = 0.048) and HR of 17.83 (95%CI: 2.02–157.11, *p* = 0.009), respectively. However, after Bonferroni correction for multiple testing none of the parameters was independently associated with overall survival (Supplementary Table S6).

**Figure 4:**
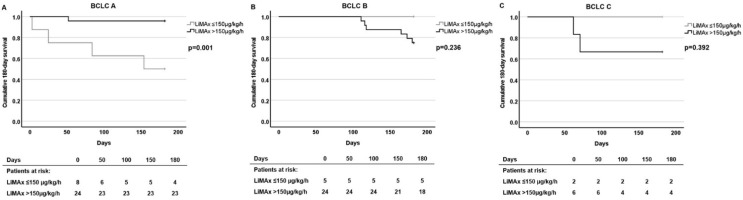
Cumulative 180-day survival in patients with different hepatocellular carcinoma stages according to the LiMAx ≤ 150 μg/kg/h and LiMAx > 150 μg/kg/h groups. Patients with (**A**) BCLC A (n = 32), (**B**) BCLC B (n = 29), and (**C**) BCLC C (n = 8) stages. Survival analyses were performed with Kaplan-Meier estimator.

Remarkably, when the patients were divided into groups according to the BCLC stage, significant differences in survival rates were detected between patients with LiMAx ≤ 150 μg/kg/h and with LiMAx > 150 μg/kg/h levels for BCLC A but not for BCLC B and C groups. In the BCLC B group, no patient died within 90 days, and in the BCLC C group, no patient died within 60 days. The 90-day survival rates of the BLCL C group were not significantly different according to the LiMAx ≤ 150 μg/kg/h and LiMAx >150 μg/kg/h groups (*p* = 0.392). In contrast, the survival rates of patients with BCLC A stage with LiMAx ≤ 150 μg/kg/h were significantly lower after 30 days (75.0 ± 15.3% vs. 100%, *p* = 0.011) and 90 days (62.5 ± 17.7% vs. 95.8 ± 4.1%, *p* = 0.011) compared to those patients with higher LiMAx levels. This was even more pronounced after 180 days for BCLC A with an estimate of 50.0 ± 17.7% vs. 95.8 ± 4.1%, *p* = 0.00; in contrast to BCLC B with an estimate of 100% vs. 75.0 ± 8.8%, *p* = 0.236; and BCLC C with an estimate of 100% vs. 66.7 ± 19.2%, *p* = 0.392, respectively (Figure 4).

In the cumulative 180-day survival data, four of the eight patients with LiMAx ≤ 150 μg/kg/h died due to liver deterioration, and only one patient with LiMAx > 150 μg/kg/h died in BCLC stage A, whereas there was no death in patients with LiMAx ≤ 150 μg/kg in BCLC stage B/C. In univariate Cox regression analysis, LiMAx results ≤ 150 μg/kg/h were associated with reduced 180-day survival (HR = 15.03 (95% CI: 1.67–134.94) *p* = 0.016) in patients with BCLC A but not with BCLC B (*p* = 0.459) and BCLC C (*p* = 0.606). In multivariate Cox regression analysis, again MELD showed an independent association with 180-day survival in BCLC stage A with a HR of 1.63 (95% CI: 1.06–2.51), *p* = 0.026), which was lost after Bonferroni correction for multiple testing (Supplementary Table S7). Finally, the overall survival rates were not different between patients with low and high LiMAx levels in the BCLC A (41.7 ± 20.5% vs. 65.9 ± 12.2%, *p* = 0.155), BCLC B (60.0 ± 21.9% vs. 53.2 ± 11.8%, *p* = 0.778) and BCLC C (50.0 ± 35.4% vs. 66.7 ± 19.2%, *p* = 0.964) groups. The median (range) survival times of patients with LiMAx ≤ 150 μg/kg/h and LiMAx > 150 μg/kg/h were in BCLC A: 12 (0–31) vs. 118 (2–34) month (*p* = 0.223), in BCLC B: 31 (11–33) vs. 14 (4–33) month (*p* = 0.106) and in BCLC C: 19 (16–21) vs. 14 (2–29) month (*p* = 0.317).

## 4. Discussion

In our study, we investigated the correlation between enzymatic liver function, based on the LiMAx test, and clinical outcome in patients with early or intermediate stage HCC who are eligible for transarterial treatments. We found that LiMAx results ≤ 150 μg/kg/h are strongly associated with decreased survival rates over 30 and 60 days, and with survival over 180 days in patients in BCLC stage A.

As liver cancer covers a wide range of stages—from very early to advanced disease, and with many treatment options from surgery to loco–regional treatments to immunotherapy—the treating physician is often faced with a great variety of therapy strategies.

In this context, measuring liver function by LiMAx might represent a useful tool in identifying patients with short survival, which was the primary aim of the study. This could be especially relevant for patients eligible to transarterial treatment with LiMAx results ≤ 150 μg/kg/h which had higher 30- and 60-day mortalities as compared to patients with LiMAx results > 150 μg/kg/h. In addition, the long-term prognosis over 180 days was significantly lower for patients in BCLC stage A with LiMAx values ≤ 150 μg/kg/h. It needs to be investigated whether those patients might have had more benefit from different treatment approaches.

Furthermore, as a secondary aim, we found no significant association of LiMAx test results with adverse events associated with transarterial treatment. Indeed, the most common side effects to TACE, prevalent in 35–100% of patients, is the post-embolization syndrome, a constellation of fever, abdominal pain, nausea, and vomiting that is transient and rather mild, and therefore potentially underreported [8,11,12,41]. Potential severe complications of TACE include liver failure, biliary or hepatic artery injury, and infection, and mortality rates from TACE are less than 2% [42]. A reason for this low rate is that main risk factors for liver failure including decompensated cirrhosis, portal vein thrombosis, large bilobar tumors, a glomerular filtration rate (GFR) less than 30 mL/min, and extra-hepatic spread are considered contraindications to TACE [43,44,45]. Similarly, severe side effects have also been rarely reported after TARE [12,41]. Therefore, our and other previous reports linking LiMAx results with side effects of transarterial treatment may have been underpowered to reliably detect associations with such rare events. Nevertheless, other studies suggested an association of LiMAx with tolerability of TACE. In fact, the only patient who had a liver failure after TACE in our cohort had a LiMAx result < 150 μg/kg/h before treatment, suggesting that a large scale study would be necessary to clarify the value of LiMAx for the prediction of adverse effects of transarterial treatment.

Several studies showed that the LiMAX test was appropriate to quantify the liver function capacity in different clinical settings of liver disease. Hence, the LiMAx test is comparable to conventional liver function tests (e.g., dynamic indocyanine green test or static tests such as bilirubin, INR, and lactate) for predicting liver function deterioration.

In some reports, LiMAx levels before TACE correlated with bilirubin and albumin levels and liver function scores, which are surrogate markers indicating liver function deteriorations after treatment [2,14,15,16,17]. However, in our study protocol we assessed liver function 4 weeks after treatment to allow for liver regeneration.

Thus, in our study, there was no association of pre-treatment LiMAx levels with changes in liver function 4 weeks after TACE/TARE. This was also not detected when the patients were divided into groups with LiMAx ≤ 150 μg/kg/h and LiMAx > 150 μg/kg/h levels according to Stockman et al. [29]. The results are in agreement with the study of Barzakova et al. [36], where the patients fully recovered one month after treatment. It seems that the LiMAx test might only be successful to detect short-term changes in liver function. Thus, in the aforementioned studies of Barzakova et al. [33] and Reichert et al. [32], the individual LiMAx levels were significantly reduced by 10% and 7% one day after TACE.

Limitations of the study were the small size of the patient cohort, the diversity of liver tumors and the large number of pretreated patients with a wide range of treatment regimen. Despite the fact that this trial was noteworthy, most of the results were not significant and several issues need to be addressed in larger follow-up studies. Furthermore, the follow-up LiMAx test was only performed in approximately 50% of patients of the initial cohort. Future multicenter studies should aim at including a broad range of patients in different tumor stages and include sequential LiMAx measurements over time.

## 5. Conclusions

In conclusion, LiMAx measurement before therapy was no appropriate predictor of the occurrence and the severity of complications 4 weeks after TACE or TARE treatment and of tumor response. However, low LiMAx levels might enable the identification of patients with poor hepatic function and decreased short-term survival after treatment, especially in early stage HCC. In view of the rapidly developing field of systemic therapies for HCC, LiMAx could play a key role in the development of personalized therapy algorithms.

## Figures and Tables

**Table 1:** Baseline patients’ characteristics.

Parameter	Overall (n = 69)	TACE (n = 57)	TARE (n = 12)	*p* Value
Age (years) ^†^	65 (48–85)	65 (48–85)	67 (56–80)	0.715
Sex (male)	54 (78.3%)	45 (78.9%)	9 (75.0%)	0.076
BMI ^†^	29.0 (19.2–52.7)	29.4 (19.2–52.7)	28.5 (23.3–41.6)	0.845
Liver cirrhosis				0.288
None Child A Child B	6 (8.7%) 47 (68.1%) 16 (23.2%)	4 (7.0%) 38 (66.7%) 15 (26.3%)	2 (16.7%) 9 (75%) 1 (8.3%)	
MELD score ^†^	8 (6–20)	9 (6–20)	8 (7–12)	0.081
ALBI score ^†^	−2.54 (−3.40–0.97)	−2.51 (−3.40–0.97)	−2.81 (−3.38–1.72)	0.182
ALBI grade				0.553
1 2 3	33 (48.5%) 33 (48.5%) 2 (2.9%)	25 (43.9%) 29 (50.9%) 2 (3.5%)	8 (66.7%) 4 (33.3%) 0	
Etiology of liver cirrhosis				0.805
Alcoholic NAFLD Viral Cryptogenic Autoimmune	38 (60.3%) 12 (19.0%) 3 (4.8%) 9 (14.3%) 1 (1.6%)	30 (52.6%) 11 (19.3%) 3 (5.3%) 8 (14.0%) 1 (1.8%)	8 (80.0%) 1 (10.0%) 1 (10.0%) 0 0	
BCLC score				0.204
A B C	32 (46.4%) 29 (42.0%) 8 (11.6%)	29 (50.9%) 22 (38.6%) 6 (10.5%)	3 (25.0%) 7 (58.3%) 2 (16.7%)	
Number of noduli				0.270
1 2 3 >3	24 (34.8%) 17 (24.6%) 11 (15.9%) 17 (24.6%)	17 (29.8%) 16 (28.1%) 10 (17.5%) 14 (24.6%)	7 (58.3%) 1 (8.3%) 1 (8.3%) 3 (25.0%)	
Largest nodule diameter (mm) ^†^	57 (9–159)	54 (12–159)	76 (9–155)	0.054
Nodules in hepatic lobe				0.671
Right Left Both	26 (37.7%) 9 (13.0%) 34 (49.3%)	20 (35.1%) 8 (14.0%) 29 (50.9%)	6 (50.0%) 1 (8.3%) 5 (41.7%)	
Repetitive TACE	17 (24.6%)	17 (24.6%)		

^†^ Median (range). The Chi-squared test was applied for categorical variables and the Mann–Whitney U-test to compare quantitative variables. ALBI: albumin–bilirubin, BCLC: Barcelona Clinic Liver Cancer score, BMI: body mass index, MELD: model for end-stage liver disease, NAFLD: nonalcoholic fatty liver disease, TACE: transarterial chemoembolization TARE: transarterial radioembolization.

**Table 2:** LiMAx results, laboratory parameters, and liver function scores before and at week 4 after transarterial treatment.

Parameter	Before TACE/TARE (n = 37)		Week 4 after TACE/TARE (n = 37)		
	Median	Range	Median	Range	*p* Value
LiMAx (µg/kg/h)	235	35–686	255 ^†^	53–604	0.397
ALT (µkat/L)	0.59	0.23–1.03	0.48	0.25–12.58	0.148
AST (µkat/L)	0.84	0.38–1.58	0.73	0.28–12.93	0.608
GGT (µkat/L)	2.66	0.42–10.53	2.23	0.58–7.45	0.837
Bilirubin (µmol/L)	18.9	4.8–87.8	16.6	4.9–70.8	0.709
Platelets (×10^9^/L)	133	51–265	127	40–240	0.778
Albumin (g/L)	40.2	28.5–48.0	38.3	21.7–48.3	0.193
INR	1.2	0.9–1.9	1.2	0.9–2.9	0.679
Creatinine (µmol/L)	79	32–124	73	29–135	0.657
ALBI score	−2.69	−3.40–1.44	−2.46	−3.39–0.92	0.181
CTP score	5	5–7	5	5–7	0.680
MELD score	8	6–18	9	6–16	0.258

The Wilcoxon signed-rank test was applied to compare the quantitative variables. ALBI: albumin–bilirubin, ALT: alanine aminotransferase, AST: aspartate aminotransferase, CTP: Child–Turcotte–Pugh, GGT: gamma–glutamyl transpeptitase, LiMAx: liver maximum function test, INR: international normalized ratio, MELD: model for end-stage liver disease, TACE: transarterial chemoembolization, TARE: transarterial radioembolization, U: unit.

## Data Availability

The data presented in this study are available on request from the corresponding author. The data are not publicly available due to legal issues.

## Supplementary Materials

The following supporting information can be downloaded at: https://www.mdpi.com/article/10.3390/cancers14215323/s1, Table S1: Changes in LiMAx, laboratory parameters and liver function scores according Child A and Child B group. Table S2: Changes in LiMAx, laboratory parameters and liver function scores according ALBI grade 1 and ALBI grade 2/3 group. Table S3: Changes in LiMAx, laboratory parameters and liver function scores according LiMAx ≤ 150 μg/kg/h and LiMAx >150 μg/kg/h levels. Table S4: Adverse events within 4 weeks after transarterial treatment. Table S5: Univariate and multivariate logistic regression analyses for the occurrence of adverse events. Table S6: Univariate and multivariate Cox regression analyses for overall survival. Table S7: Univariate and multivariate Cox regression analyses for 180-day survival in patients with BCLC stage A.

## Abbreviations

ACLF: acute-on-chronic-liver-failure, ALBI: albumin–bilirubin, ALT: alanine aminotransferase, AST: aspartate aminotransferase, BCLC: Barcelona Clinic Liver-Cancer score, BMI: body mass index, CI: confidence interval, CLIP: Cancer of the Italian Liver Program, CTP: Child-Turcotte-Pugh, GBq: Giga-Becquerel, GFR: glomerular filtration rate, GGT: gamma–glutamyl transpeptitase, HCC: hepatocellular carcinoma, HR: hazard ratio, INR: international normalized ratio, LiMAx: liver maximum capacity test, MELD: model for end-stage liver disease, NAFLD: nonalcoholic fatty liver disease, OR: odds ratio, PTHF: post-TACE hepatic liver failure, RC: regression coefficient, REF: reference, REILD: radioembolization-induced liver disease, SE: standard error, SIR: Society of Interventional Radiology, SR: survival rate, TACE: transarterial chemoembolization, TARE: transarterial radioembolization
