# Supplementary material for: The Liver Maximum Capacity Test (LiMAx) Is Associated with Short-Term Survival in Patients with Early Stage HCC Undergoing Transarterial Treatment
Source: Cancers (Basel). 2022 Oct 28;14(21):5323. doi: 10.3390/cancers14215323 (PMC9657475; doi:10.3390/cancers14215323)
Supplement: Supplementary file 1 [file cancers-14-05323-s001.zip › cancers-1922154-supplementary.pdf]

Supplementary Materials

The liver maximum function capacity test (LiMAx) is associated with short-term survival in patients with early stage HCC undergoing transarterial treatment

Janett Fischer, Stella Wellhöner, Sebastian Ebel, Thomas Lincke, Albrecht Böhlig, Florian Gerhardt, Rhea Veelken, Holger Gößmann, Karen Geva Steinhoff, Timm Denecke, Osama Sabri, Thomas Berg, Florian van Bömmel

Table of contents

Supplementary Results

Table S1 2

Table S2 3

Table S3 4

Table S4 5

Table S5 5

Table S6 6

Table S7 6

Table S1: Changes in LiMAx, laboratory parameters and liver function scores according Child A and Child B group. All values presented as median (range).

Parameter	Child A (n=29) Before TACE/TARE	Child A (n=29) After TACE/TARE	Child A (n=29) p value	Child B (n=5) Before TACE/TARE	Child B (n=5) After TACE/TARE	Child B (n=5) p value	Child A vs. Child B (p value) Before TACE/TARE	Child A vs. Child B (p value) After TACE/TARE
LiMAx (µg/kg/h)	229 (87-686)	261 (53-604)	0.018	181 (35-282)	84 (60-228)	0.625	0.292	0.010
ALT (µkat/L)	0.58 (0.23-1.03)	0.46 (0.25-12.58)	0.081	0.63 (0.52-0.68)	0.59 (0.25-0.79)	1.000	0.370	0.227
AST (µkat/L)	0.74 (0.37-12.70)	0.71 (0.28-12.93)	0.250	1.12 (0.61-1.58)	1.26 (0.49-1.56)	0.625	0.026	0.092
GGT (µkat/L)	2.39 (0.42-6.95)	2.17 (0.58-7.45)	0.571	2.99 (1.43-10.53)	2.90 (0.90-6.09)	0.125	0.444	0.737
Bilirubin (µmol/L)	13.8 (4.8-34.1)	16.6 (5.5-44.6)	0.421	17.9 (7.1-87.8)	35.2 (6.9-70.8)	0.625	0.154	0.055
Platelets ($x10^9$/L)	133 (51-221)	120 (40-240)	0.741	109 (74-144)	123 (74-145)	1.000	0.611	0.780
Albumin (g/L)	40.9 (31.7-46.6)	40.4 (21.7-47.0)	0.003	36.1 (28.5-39.5)	31.2 (25.2-36.1)	0.063	0.011	0.004
INR	1.2 (0.9-1.9)	1.2 (0.9-1.9)	0.717	1.2 (1.0-1.5)	1.3 (1.0-1.4)	1.000	0.698	0.383
Creatinine (µmol/L)	77 (32-93)	70 (29-109)	0.577	86 (61-124)	73 (60-135)	1.000	0.167	0.300
ALBI score	-2.76 (-3.38--1.77)	-2.58 (-3.20--0.92)	0.0007	-1.95 (-2.53--1.44)	-1.87 (-1.99--1.25)	0.125	0.008	0.0008
CTP score	5 (5-6)	5 (5-7)	0.563	7 (7-7)	7 (7-7)	1.000	$3.95x10^{-6}$	$2.16x10^{-5}$
MELD score	8 (6-15)	8 (6-15)	0.243	10 (8-18)	13 (10-16)	0.813	0.027	0.003

The Mann–Whitney U-test and Wilcoxon signed-rank test were applied to compare the quantitative variables. ALT: alanine aminotransferase, AST: aspartate aminotransferase, CTP: Child-Turcotte-Pugh, GGT: gamma-glutamyl transpeptitase, LiMAx: liver maximum capacity test, INR: international normalized ratio, TACE: transarterial chemoembolization, TARE: transarterial radioembolization

Table S2: Changes in LiMAx, laboratory parameters and liver function scores according ALBI grade 1 and ALBI grade 2/3 group. All values presented as median (range).

	ALBI grade 1 (n=22)			ALBI grade 2/3 (n=14)			ALBI grade 1 vs. ALBI grade 2/3 (p value)	
**Parameter**	**Before TACE/TARE**	**After TACE/TARE**	**p value**	**Before TACE/TARE**	**After TACE/TARE**	**p value**	**Before TACE/TARE**	**After TACE/TARE**
LiMAx (µg/kg/h)	276 (156-686)	316 (53-604)	0.168	173 (35-282)	192 (60-283)	0.414	0.0001	0.0002
ALT (µkat/L)	0.55 (0.23-1.01)	0.42 (0.25-12.58)	0.468	0.63 (0.38-1.03)	0.54 (0.25-0.79)	0.185	0.090	0.178
AST (µkat/L)	0.62 (0.38-1.07)	0.55 (0.28-12.93)	0.962	0.96 (0.56-1.58)	0.79 (0.49-1.56)	0.223	0.0001	0.016
GGT (µkat/L)	2.14 (0.44-5.21)	1.93 (0.71-7.45)	0.732	2.95 (0.44-10.53)	3.05 (0.58-7.02)	0.122	0.299	0.243
Bilirubin (µmol/L)	19.7 (4.8-23.2)	14.4 (4.9-44.6)	0.156	21.6 (7.1-87.8)	21.5 (6.9-70.8)	0.867	0.0008	0.002
Platelets ($x10^9$/L)	149 (51-265)	138 (40-225)	0.168	102 (55-221)	108 (58-240)	0.021	0.130	0.555
Albumin (g/L)	43.4 (39.0-48.0)	42.7 (236.2-48.3)	0.109	36.0 (28.5-39.5)	34.5 (21.7-37.1)	0.003	$6.06x10^{-9}$	$6.85x10^{-9}$
INR	1.1 (0.9-1.4)	1.1 (0.9-1.9)	0.546	1.2 (1.0-1.9)	1.3 (1.0-1.6)	0.797	0.088	0.058
Creatinine (µmol/L)	79 (48-95)	72 (40-109)	0.231	78 (32-124)	71 (29-135)	0.841	0.892	0.917
ALBI score	-2.96 (-3.40--2.63)	-2.85 (-3.39--2.25)	0.033	-2.25 (-2.53--1.44)	-1.97 (-2.39--0.92)	0.004	$5.27x10^{-10}$	$2.11x10^{-9}$
CTP score	5 (5-6)	5 (5-7)	0.312	6 (5-7)	5 (5-7)	1.000	0.002	0.121
MELD score	8 (6-10)	8 (6-15)	0.073	10 (7-18)	11 (7-16)	0.920	0.002	0.014

The Mann–Whitney U-test and Wilcoxon signed-rank test were applied to compare the quantitative variables. ALT: alanine aminotransferase, AST: aspartate aminotransferase, CTP: Child-Turcotte-Pugh, GGT: gamma-glutamyl transpeptitase, LiMAx: liver maximum capacity test, INR: international normalized ratio, TACE: transarterial chemoembolization, TARE: transarterial radioembolization

Table S3: Changes in LiMAx, laboratory parameters and liver function scores according LiMAx ≤150 µg/kg/h and LiMAx >150 µg/kg/h levels. All values presented as median (range).

Parameter	LiMAx ≤150 µg/kg/h (n=5)			LiMAx >150 µg/kg/h (n=31)			LiMAx ≤150 vs. LiMAx >150 µg/kg/h (p value)	
	Before TACE/TARE	After TACE/TARE	p value	Before TACE/TARE	After TACE/TARE	p value	Before TACE/TARE	After TACE/TARE
LiMAx (µg/kg/h)	92 (35-115)	165 (60-261)	0.063	266 (156-686)	272 (53-604)	0.223	$8.60x10^{-7}$	0.028
ALT (µkat/L)	0.65 (0.53-0.68)	0.51 (0.41-0.75)	0.438	0.56 (0.23-1.03)	0.46 (0.25-12.58)	0.301	0.143	0.449
AST (µkat/L)	1.09 (0.73-1.58)	0.78 (0.72-1.53)	0.613	0.81 (0.38-1.17)	0.64 (0.28-12.93)	0.758	0.021	0.122
GGT (µkat/L)	1.97 (0.42-3.54)	2.06 (0.58-6.95)	0.316	2.90 (0.38-10.53)	2.23 (0.71-7.44)	0.413	0.261	0.554
Bilirubin (µmol/L)	34.1 (31.4-87.8)	30.9 (15.5-70.8)	0.438	13.5 (4.8-26.0)	15.6 (4.9-44.6)	0.117	$2.64x10^{-6}$	0.002
Platelets ($x10^9$/L)	89 (55-180)	92 (53-173)	1.000	140 (51-265)	136 (40-240)	0.637	0.162	0.229
Albumin (g/L)	35.7 (31.9-38.3)	34.7 (21.7-37.9)	0.063	41.0 (28.5-48.0)	40.6 (25.2-48.3)	0.007	0.006	0.010
INR	1.3 (1.1-1.8)	1.3 (1.1-1.6)	0.500	1.1 (0.9-1.9)	1.1 (0.9-1.9)	0.470	0.041	0.116
Creatinine (µmol/L)	81 (32-109)	80 (29-110)	0.500	79 (48-124)	71 (40-135)	0.335	0.976	0.849
ALBI score	-2.01 (-2.26--1.44)	-1.91 (-2.44--0.92)	0.063	-2.83 (-3.40--1.61)	-2.71 (-3.39--1.25)	0.003	$4.40x10^{-4}$	0.001
CTP score	6 (5-7)	5 (5-7)	0.500	5 (5-7)	5 (5-7)	0.359	0.095	0.763
MELD score	13 (8-18)	13 (8-16)	0.312	8 (6-15)	9 (6-15)	0.059	0.001	0.021

The Mann–Whitney U-test and Wilcoxon signed-rank test were applied to compare the quantitative variables. ALT: alanine aminotransferase, AST: aspartate aminotransferase, CTP: Child-Turcotte-Pugh, GGT: gamma-glutamyl transpeptitase, LiMAx: liver maximum capacity test, INR: international normalized ratio, TACE: transarterial chemoembolization, TARE: transarterial radioembolization.

Table S4. Adverse events within 4 weeks after transarterial treatment.

Adverse effect	Number (%)	
	**TARE (n=12)**	**TACE (n=57)**
Mental confusion	0	2 (3.5%)
Fatigue	0	8 (14.0%)
Epigastric pressure	2 (16.7%)	3 (5.3%)
Abdominal fullness	0	2 (3.5%)
Flatulence	0	3 (5.3%)
Diarrhea	0	3 (5.3%)
Pruritus	2 (16.7%)	2 (3.5%)
Ascites		
Mild to moderate	1 (8.3%)	2 (3.5%)
Severe	0	5 (8.8%)
Hepatic encephalopathy	0	2 (3.5%)
Kidney failure	0	2 (3.5%)
Liver failure	0	1 (1.8%)
Myocardial infarct		1 (1.8%)
Death		2 (3.5%)
Lost to follow up		1 (1.8%)

Table S5: Univariate and multivariate logistic regression analyses for the occurrence of adverse events.

Parameter	Univariate analysis				Multivariate analysis			
	**RC**	**SE**	**OR (95% CI)**	**p value**	**RC**	**SE**	**OR (95% CI)**	**p value**
LiMAx ≤150 µg/kg/h	1.197	0.702	3.31 (0.84-13.11)	0.088	1.240	1.210	3.46 (0.32-37.27)	0.307
Male sex	0.367	0.590	1.44 (0.45-4.59)	0.534			-	
Age (years)	0.020	0.028	1.02 (0.98-1.07)	0.474			-	
CTP score	0.776	0.357	**2.15 (1.07-4.33)**	**0.032**	0.452	0.460	1.57 (0.64-3.84)	0.320
MELD	0.121	0.082	1.13 (0.93-1.33)	0.143				
ALBI score	1.005	0.508	**2.73 (1.01-7.40)**	**0.048**	-1.539	5.040	0.22 (0-4165.97)	0.760
ALT (µkat/L)	1.043	0.912	2.84 (0.48-16.94)	0.252			-	
AST (µkat/L)	1.762	0.858	**5.83 (1.08-31.98)**	**0.040**	1.460	1.310	4.31 (0.33-55.97)	0.264
GGT (µkat/L)	-0.970	0.115	0.91 (0.72-1.14))	0.400			-	
Bilirubin (µmol/L)	0.041	0.021	1.04 (1.00-1.09)	0.050	0.002	0.070	1.00 (0.87-1.16)	0.973
Platelets (x10^9/L)	-0.002	0.004	1.00 (0.99-1.01)	0.641			-	
Albumin (g/L)	-0.092	0.054	0.91 (0.82-1.01)	0.085	-0.122	0.450	0.89 (0.37-2.14)	0.788
INR	1.239	1.216	3.45 (0.32-37.44))	0.308			-	
Creatinine (µmol/L)	-0.003	0.009	1.00 (0.98-1.01)	0.738			-	
BCLC Score								
A			REF				-	
B	-0.118	0.521	0.89 (0.32-2.47)	0.821			-	
C	1.620	1.129	5.06 (0.55-46.24)	0.151			-	
Treatment								
TACE	0.099	0.646	1.10 (0.31-3.92)	0.878			-	
TARE			REF				-	

ALBI: albumin-bilirubin, ALT: alanine aminotransferase, AST: aspartate aminotransferase, BCLC: Barcelona Clinic Liver Cancer score, CI: confidence interval, CTP: Child-Turcotte-Pugh, GGT: gamma-glutamyl transpeptitase, LiMAx: liver maximum capacity test, HR: hazard ratio, INR: international normalized ratio,

MELD: model for end-stage liver disease, RC: regression coefficient, REF: reference, SE: standard error, TACE: transarterial chemoembolization, TARE: transarterial radioembolization

Table S6: Univariate and multivariate Cox regression analyses for overall survival.

Parameter	Univariate analysis				Multivariate analysis			
	RC	SE	HR (95% CI)	p value	RC	SE	HR (95% CI)	p value
LiMAx ≤150 µg/kg/h	0.502	0.430	1.65 (0.71-3.84)	0.243			-	
Male sex	0.147	0.500	1.16 (0.44-3.09)	0.769			-	
Age (years)	-0.047	0.026	0.95 (0.91-1.01)	0.074	-0.015	0.030	0.99 (0.93-1.04)	0.605
CTP Child A			REF				REF	
Child B	1.070	0.424	**2.93 (1.27-6.76)**	**0.012**	0.317	0.582	1.37 (0.44-4.30)	0.586
MELD	0.161	0.058	**1.17 (1.05-1.31)**	**0.005**	**0.142**	**0.072**	**1.15 (1.00-1.33)**	**0.048**
ALBI score	1.120	0.368	**3.10 (1.51-6.37)**	**0.002**				
Grade 1			REF				REF	
Grade 2	0.950	0.458	**2.58 (1.05-6.34)**	**0.038**	0.133	0.557	1.14 (0.38-3.41)	0.812
Grade 3	3.921	0.932	**50.43 (8.12-313.06)**	**$2.57x10^{-5}$**	**2.881**	**1.110**	**17.83 (2.02-157.11)**	**0.009**
BCLC Score								
A			REF				-	
B	0.160	0.428	1.17 (0.51-2.71)	0.708			-	
C	0.219	0.652	1.25 (0.35-4.47)	0.737			-	
Treatment								
TACE	-0.547	0.512	0.58 (0.21-1.58)	0.264			-	
TARE			REF				-	

ALBI: albumin-bilirubin, BCLC: Barcelona Clinic Liver Cancer score, CI: confidence interval, CTP: Child-Turcotte-Pugh, LiMAx: liver maximum capacity test, HR: hazard ratio, ratio, MELD: model for end-stage liver disease, RC: regression coefficient, REF: reference, SE: standard error, TACE: transarterial chemoembolization, TARE: transarterial radioembolization

Table S7: Univariate and multivariate Cox regression analyses for 180-day survival in patients with BCLC stage A.

Parameter	Univariate analysis				Multivariate analysis			
	RC	SE	HR (95% CI)	p value	RC	SE	HR (95% CI)	p value
LiMAx ≤150 µg/kg/h	2.710	1.120	**15.03 (1.67-134.94)**	**0.016**	0.608	1.786	1.84 (0.01-60.85)	0.733
Male sex	3.652	3.969	38.57 (0.02-92240)	0.357			-	
Age (years)	-0.094	0.063	0.91(0.80-1.03)	0.136			-	
CTP Child A			REF				-REF	
Child B	2.667	1.120	**14.39 (1.60-129.21)**	**0.017**	2.425	1.657	11.30 (0.44-290.58)	0.143
MELD	0.405	0.134	**1.50 (1.15-1.95)**	**0.003**	**0.489**	**0.219**	**1.63 (1.06-2.51)**	**0.026**
ALBI score	2.767	0.998	**15.91 (2.25-112.45)**	**0.006**	1.899	1.911	6.68 (0.16-282.63)	0.320
Grade 1			REF					
Grade 2	11.330	171.011	0	0.947			-	
Grade 3	13.881	171.012	0	0.935			-	
Treatment								
TACE	3.161	6.540	23.61 (0-870987)	0.629			-	
TARE			REF				-	

ALBI: albumin-bilirubin, BCLC: Barcelona Clinic Liver Cancer score, CI: confidence interval, CTP: Child-Turcotte-Pugh, LiMAx: liver maximum capacity test, HR: hazard ratio, MELD: model for end-stage liver disease, RC: regression coefficient, REF: reference, SE: standard error, TACE: transarterial chemoembolization, TARE: transarterial radioembolization
